# Supplementary material for: Mental health service utilization in a low resource setting: A qualitative study on perspectives of health professionals in Northwest Ethiopia
Source: PLoS One. 2022 Nov 28;17(11):e0278106. doi: 10.1371/journal.pone.0278106 (PMC9704635; doi:10.1371/journal.pone.0278106)
Supplement: S1 File — (DOCX) [file pone.0278106.s001.docx]

**Mental health service utilization in a low resource setting: A qualitative study on perspectives of health professionals in Northwest Ethiopia**

Tesfa Mekonen^1,2,3^, Gary C.K. Chan^2^, Tilahun Belete^3^, Melak Menberu^3^, Lily Davidson^1^, Leanne Hides^1,2^, Janni Leung^1,2^

^1^School of Psychology, The University of Queensland, Australia

^2^National Centre for Youth Substance Use Research, The University of Queensland, Australia

^3^Psychiatry Department, Bahir Dar University, Ethiopia

**Semi-structured interview guide**

| 1. General information about the participant |
| --- |
| *Can you please tell me about yourself?*   - Age - Sex - Profession (Nurse, HO, Midwife, mental health professional, GP, internist,………) - Position (department head, facility head…) - Facility type (health center, primary hospital, general hospital, specialized hospital…) |
| 1. Experience related to mental health |
| 1. **What is your experience related to mental illness?** |
| *Likely prompts:*   - *Do you know anyone with mental illness?* - *Have you ever treated a patient with mental illness?* - *What was the experience like?* - *Do you think you will treat patients with mental illness in the future?* |
| 1. **What do you think about the treatment of mental illness and its effectiveness?** |
| *Likely prompts:*   - *How difficult or easy is it to treat patients with mental illness?* - *Does treatment help?* - Consideration of mental health service in annual plans? |
| 1. Help-seeking |
| 1. **What do you think about help seeking behavior of people with mental illness?** |
| *Likely prompts:*   - *Medical help?* - *Untreated duration?* - *Service accessibility/availability?* - *Adherence to treatment?* |
| 1. Barriers to seek professional help |
| 1. **What do you think could be the main barriers for people with mental disorders to come to health facilities for treatment?** |
| *Likely prompts:*   - *At the individual level (e.g. fear of stigma?)* - *At the community level (e.g. culture?)* - *At health facility level (e.g. cost, distance? service availability? professionals attitude?)* - *Mental health literacy?* - *What beliefs about mental illness have you noticed that might prevent people from getting treatment?* |
| 1. **What could be done to improve access to mental health care and overcome these barriers?** |
| *Likely prompts:*   - *individual level (e.g. fear of stigma?)* - *community? (stigma?)* - *health professionals? (attitude? In-service training?)* - *Government? (leadership, free treatment, decentralization, campaign?)* |
| 1. Detection of people with mental illness |
| 1. **How difficult or easy is it to recognize (detect) mental illness?** |
| *Likely prompts:*   - *By the patient themselves* - *By the community members* - *In the health facilities (diagnosis)* |
| 1. **How could detection of mental illness be improved?** |
| *Likely prompts:*   - *Who can help in the detection of mental illness?* - *in the community?* - *in health facilities? (trained manpower?)* |
| 1. Additional comments |
| **Do you have anything else to add?** |
| Thank you! |
