# Supplementary material for: Mental health service utilization in a low resource setting: A qualitative study on perspectives of health professionals in Northwest Ethiopia
Source: PLoS One. 2022 Nov 28;17(11):e0278106. doi: 10.1371/journal.pone.0278106 (PMC9704635; doi:10.1371/journal.pone.0278106)
Supplement: Attachment — Submitted filename: Response to reviewer_R2.docx [file pone.0278106.s004.docx]

**Response to editor’s comment**

Dear Editor,

Thank you for your comments and suggestions. We have revised the manuscript accordingly and we provided the Standards for reporting qualitative research (SRQR) checklist supplementary file.

**Editor:**

1. Upload as a supplementary file a checklist explaining how your study addresses and fulfills the criteria for qualitative studies, using one of the following checklists:
the Consolidated criteria for reporting qualitative research (COREQ) checklist or Standards for reporting qualitative research (SRQR) checklist.
Further reporting guidelines can be found in the Equator Network's Guidelines for reporting qualitative research.
You can find the links to the above here, under the heading of Qualitative Studies: <https://journals.plos.org/plosone/s/submission-guidelines#loc-qualitative-research>

**[Response]:** Thank you, we used the Standards for reporting qualitative research (SRQR) to guide our writing (Page 8, Line 181 & 182). The Standards for reporting qualitative research (SRQR) checklist is uploaded as supplementary file. The caption for the supplementary file is also included at the end of the manuscript (Page 23).

2. Cross-check again the language used to make sure the manuscript does not include fundamental orthographic and expression errors.

**[Response]:** Thank you, the manuscript is proofread for potential errors, revised, and edited. We provided the track changes of the edits in the marked-up copy of the manuscript

**Thank you!**
